# Coexistence of GH-Producing Pituitary Macroadenoma and Meningioma in a Patient with Multiple Endocrine Neoplasia Type 1 with Hyperglycemia and Ketosis as First Clinical Sign

**DOI:** 10.1155/2017/2390797

**Published:** 2017-11-01

**Authors:** A. Herrero-Ruiz, H. S. Villanueva-Alvarado, J. J. Corrales-Hernández, C. Higueruela-Mínguez, J. Feito-Pérez, J. M. Recio-Cordova

**Affiliations:** ^1^Service of Endocrinology and Nutrition, University Clinical Hospital of Salamanca, Paseo de San Vicente No. 58, 37007 Salamanca, Spain; ^2^Department of Medicine, University of Salamanca, Campus Miguel de Unamuno, s/n, 37007 Salamanca, Spain; ^3^Cancer Research Institute (IBMCC-CSIC/USAL) and Institute for Biomedical Research, University of Salamanca, Salamanca, Spain; ^4^Service of Anatomic Pathology, University Clinical Hospital of Salamanca, Paseo de San Vicente No. 58, 37007 Salamanca, Spain

## Abstract

We present the clinical case of a patient who was admitted with an onset of diabetes mellitus (DM) with associated ketosis and whose clinical, hormonal, and radiological evolution revealed the presence of primary hyperparathyroidism, pancreatic neuroendocrine tumor, and GH-producing pituitary macroadenoma in the context of multiple endocrine neoplasia type 1 (MEN1). DM is relatively common in cases of acromegaly, but it is not generally associated with ketosis. Simultaneously, the patient presented a meningioma, which is associated with pituitary macroadenoma only in extremely rare cases.

## 1. Introduction

Multiple endocrine neoplasia type 1 (MEN1) is an autosomal dominant syndrome characterized by the combined appearance of tumors in the parathyroid glands, pancreas islet cells, and the anterior pituitary. This type of syndromes is known to be associated with the secretion of a wide range of hormones which are often responsible for alterations in the glucose metabolism. However, the presence of diabetic ketosis and/or ketoacidosis is a rare clinical situation, with very few cases reported so far. Also, and in an even less common case, the patient simultaneously presented a GH-producing pituitary macroadenoma and a meningioma, a combination which is extremely rare.

## 2. Case Report

We present the clinical case of a 35-year-old woman with a history of Chagas disease who was admitted in the Unit of Endocrinology with hyperglycemia and ketosis in the context of onset of DM with weakness, polydipsia, and polyuria of 4 months of evolution, together with a weight loss of 10 kg over the last 6 months. Also, she presented amenorrhea for 4 months and hyperhidrosis. The examination revealed a slight prognathism, growth of acral parts of the body, and grade 1 goiter with a 2-cm left thyroid nodule.

The analysis showed glucose 248 mg/dL, HbA1c 14.6%, calcium 11.3 mg/dL, phosphorus 2.3 mg/dL, and urine calcium 513 mg/24 h. Given the initial findings, the study was expanded to include a hormone profile test (Table 1), a thyroid ultrasound (28 × 16-mm mixed nodule in the left lobe and 1.2 × 0.9 cm hypoechoic nodule in the right infrathyroid region, which suggests an enlarged parathyroid gland), and an ultrasound-guided fine-needle aspiration (UGFNA) of the left thyroid nodule compatible with benign follicular nodule.

**Figure 1:**
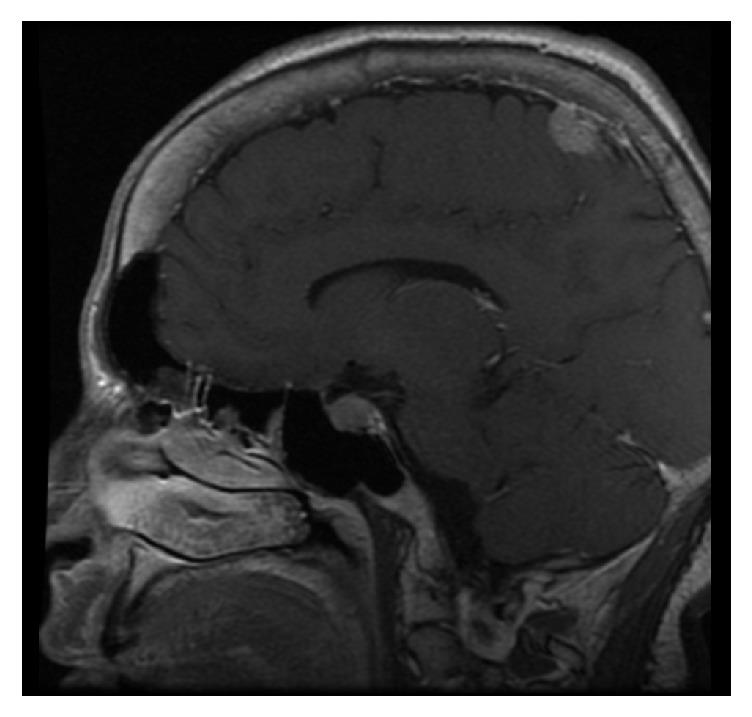
(NMR) pituitary macroadenoma and left superior parietal extra-axial lesion compatible with meningioma.

The endocrine study confirmed the clinical suspicion of primary hyperparathyroidism and GH hypersecretion. The parathyroid SPECT/CT was compatible with right parathyroid adenoma. A nuclear magnetic resonance (NMR) revealed a pituitary macroadenoma of 20 × 13 × 15 mm which spread to the right cavernous sinus, with displacement of the optic chiasm, and a left superior extra-axial parietal lesion of 20 × 20 × 12 mm which suggested a meningioma (Figure 1).

**Figure 2:**
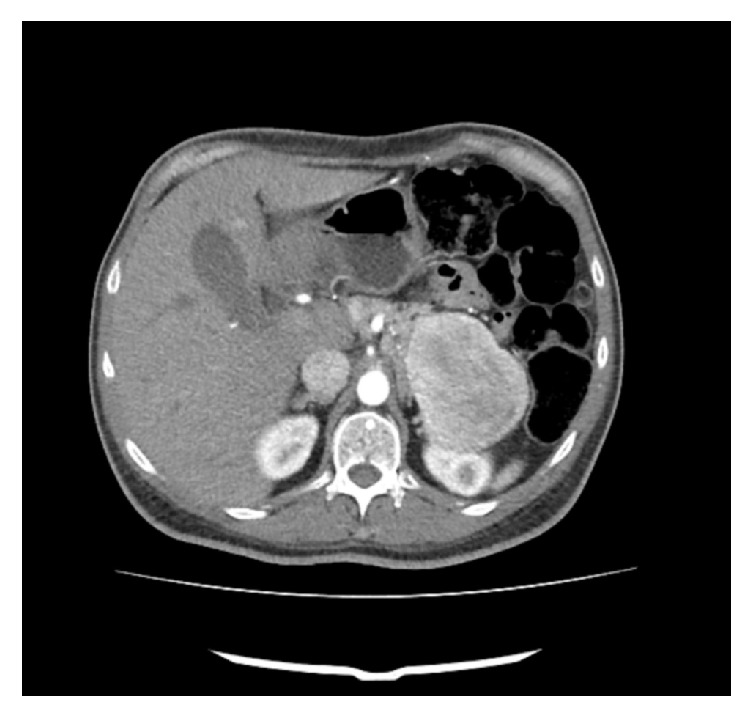
(CT) tail of pancreas with hypervascular heterogeneous and well-defined mass with lobulated edges measuring 6.8 × 7.7 × 6.4 cm.

Given these findings and the clinical suspicion of MEN1, the study was completed with a CT scan of the neck, chest, abdomen, and pelvis, which showed a hypervascular heterogeneous mass with lobulated and well-defined edges of 6.8 × 7.7 × 6.4 cm in the tail of pancreas, plus another mass of similar characteristics of 6 × 4.2 cm in the uncinate process and at least three more pancreatic focal lesions of less than 1 cm on the head and neck of pancreas (Figure 2). In the liver there were several hypervascular lesions in segments II and III, of 0.9 cm, compatible with metastatic involvement. Tumor markers revealed increased levels of somatostatin (30.9 pmol/L), pancreatic polypeptide (>200 pmol/L), and calcitonin (9.6 pg/mL), with normal levels of chromogranin A, gastrin, glucagon, and vasoactive intestinal polypeptide.

**Figure 3:**
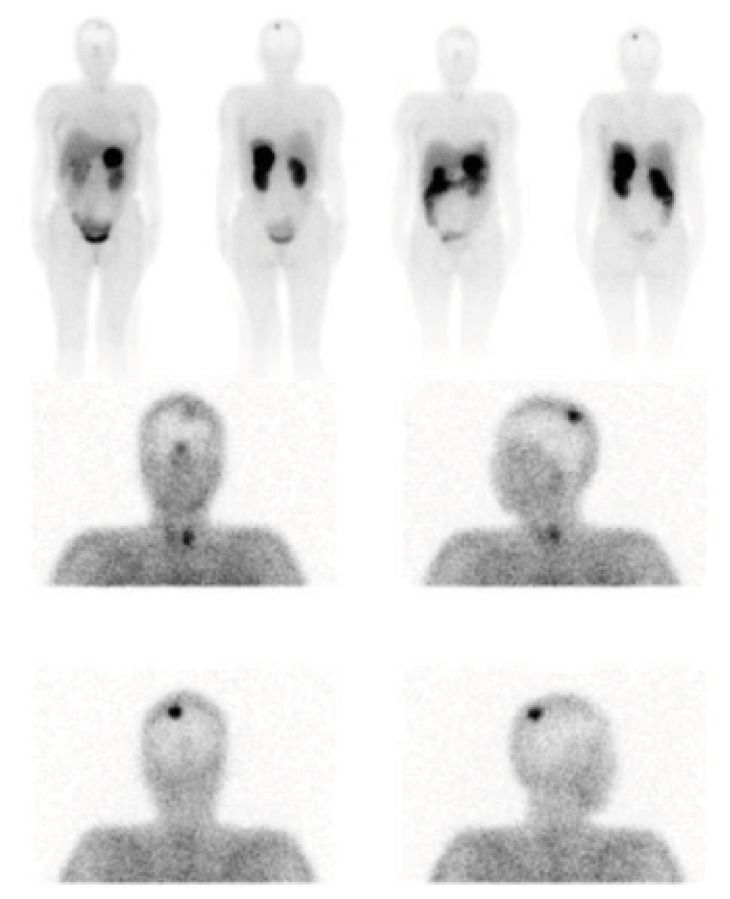
OctreoScan showing a pathological deposit at the upper left parietal level caused by meningioma and in the tail of pancreas.

A somatostatin receptor scintigraphy (OctreoScan) was performed showing a pathological deposit in the tail of pancreas which suggested a tumor with expression of somatostatin receptors and also at the upper left parietal level, which was caused by a meningioma (Figure 3). The endoscopic ultrasound-guided fine-needle aspiration biopsy of the mass in the pancreas was compatible with a neuroendocrine tumor (NET). A genetic study confirmed the clinical suspicion of MEN1. The patient was a heterozygous carrier of the pathogenic change c.1378C>T (p. Arg460^*∗*^).

The patient started treatment with high doses of basal-bolus insulin therapy, somatostatin analogs, and cinacalcet. Afterwards, cabergoline was added due to the persistence of high levels of IGF1. The pituitary macroadenoma was resected through a transnasal transsphenoidal and the IGF1 levels went back to normal, with octreotide treatment. The anatomic pathology revealed a pituitary somatotroph adenoma (densely granulated) and the immunohistochemical study: CAM 5.2 (+++), GH (+++), p53 (−), and MIB-1 <1%. An abdominal NMR was performed to control the evolution of her condition, and it still showed two heterogeneous lesions on the head and tail of pancreas, of 5.5 × 4.5 × 4 cm and 6 × 7.5 × 8 cm, respectively, contrast-enhanced and with well-defined edges. Also, the liver had normal size, morphology, and intensity, and the image did not reveal the lesions described in the CT scan image.

**Figure 4:**
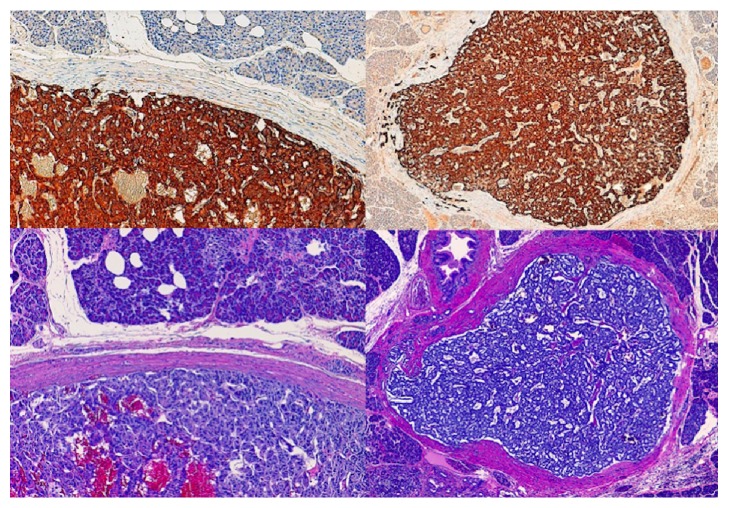
Immunohistochemistry with positive cells for glucagon.

After an assessment by an interdisciplinary board, the patient underwent total pancreaticoduodenectomy, cholecystectomy, and splenectomy. The anatomical pathology showed 7 well-differentiated (G1) NETs in the pancreas, 1 well-differentiated NET (G1) in the pylorus of <0.5 cm, 14 lymph nodes in the tail of pancreas with no sign of malignancy, and 2 lymph nodes with NET metastasis out of 12 isolated nodes on the head of pancreas (pT2N1). All the tumors expressed chromogranin A and synaptophysin, with proliferation mediated by Ki67 <1%, except for the node found in the neck of pancreas, in which it reached 2-3%. Also, 2 out of the 7 tumors were intensely positive for glucagon (100%) (Figure 4), and an additional node in the body of pancreas was positive for calcitonin (70%), with positive isolated cells for glucagon and somatostatin (<2%).

## 3. Discussion

We present the case of a patient with MEN1 with some peculiar features.

(1) DM with ketosis as its first manifestation: alterations in the metabolism of glucose are a common characteristic in acromegaly, with a described prevalence of DM ranging from 19 to 56% [1–3]. However, these alterations are generally due to the insulin resistance caused by an excess of GH and IGF1, with an increase of gluconeogenesis and a decrease of peripheral glucose uptake [4, 5], and it typically does not show a tendency to ketosis, with only 11 cases of ketoacidosis having been described as the first sign of acromegaly [4]. Some authors have analyzed the factors which may predispose to alterations in the glucose metabolism of these patients, and they are related to the levels of IGF1 [1], GH [6], age, body mass index, arterial hypertension, and time of evolution of the disease [2, 3, 6]. In our case, hyperprolactinemia, due to the compression of the pituitary stalk, may have also contributed to the alteration in glucose metabolism through an increase in insulin resistance. Some of the mechanisms suggested to explain this include a decrease in insulin receptors and/or deficiencies at a postreceptor level [4]. McCallum et al. identified an increased prevalence of diabetes and glucose intolerance in patients with MEN1, and several theories have been put forward in which adiponectin and enteropancreatic markers might be involved, or the MEN1 gene, which may cause a predisposition to this resistance [7]. On the other hand, primary hyperparathyroidism has also been associated with an increase in insulin resistance and DM [7]. Therefore, there are several underlying mechanisms which may have contributed to the atypical clinical presentation of our patient.

(2) Another peculiar trait in our case is the concomitant presence of a pituitary tumor and meningioma, because this is a rare clinical situation, with only 33 cases described [8, 9]. Meningiomas represent 15–25% of all intracranial neoplasms, with an annual incidence of 6 per 100.000 people [8–11]. Radiotherapy is known to play a role in the appearance of intracranial tumors, but in cases such as ours, without prior exposition to radiotherapy, the origin is still unclear. Some authors suggest that it may happen by sheer chance, whereas others describe theories that may explain this association [8, 10]. Suzuki et al. suggest an involvement of the activation of signaling pathways for the receptor tyrosine kinases [12], and Friend et al. showed that meningioma may express GH and IGF1 receptors [13]. Although there are cases described in the literature of an association of meningioma with functioning and nonfunctioning pituitary adenomas, in the case of functioning adenomas, GH-producing varieties seem to predominate [9, 10, 14]. It remains to be seen whether GH itself or an overexpression of IGF1 receptors in these tumors induces a transformation into a meningioma. On the other hand, Asgharian et al. proved in a prospective study of 74 patients with MEN1 that 8% of the patients developed meningioma after 18 years of follow-up, and it is believed that the alterations in the MEN1 gene may have participated in its pathogenesis [15].

## Figures and Tables

**Table 1:** Hormone measurements in plasma.

Hormone	Value	Normal range
TSH (*µ*UI/mL)	4.7	0.27–4.2
Free T4 (ng/dL)	1.1	0.82–1.78
FSH (mUI/mL)	3.3	3.5–12.5
LH (mUI/mL)	1.9	2.4–12.6
Estradiol (pg/mL)	<5	12.5–166
Prolactin (ng/mL)	59.2	4.79–23.3
GH (ng/mL)	48.1	0–8
IGF1 (ng/mL)	702	109–284
Cortisol (*µ*g/dL)	10.5	2.69–18
PTH (pg/mL)	203.4	11–67
